# Interactions between neutrophils and non-small cell lung cancer cells: enhancement of tumor proliferation and inflammatory mediator synthesis

**DOI:** 10.1007/s00262-014-1606-z

**Published:** 2014-09-04

**Authors:** Katja Hattar, Katharina Franz, Michael Ludwig, Ulf Sibelius, Jochen Wilhelm, Jürgen Lohmeyer, Rajkumar Savai, Florentine S. B. Subtil, Gabriele Dahlem, Bastian Eul, Werner Seeger, Friedrich Grimminger, Ulrich Grandel

**Affiliations:** 1 Department of Internal Medicine IV/V, University of Giessen and Marburg Lung Center (UGMLC), Klinikstrasse 33, Giessen, Germany; 2 Department of Anaesthesiology, University of Giessen, Giessen, Germany; 3 Department of Internal Medicine II, Biostatistics Group, University of Giessen and Marburg Lung Center (UGMLC), Giessen, Germany; 4 Department of Radiotherapy and Radiooncology, Philipps-University, Marburg, Germany; 5 Max-Planck Institute for Heart and Lung Research, Bad Nauheim, Germany; 6 Department of Internal Medicine II, University of Giessen and Marburg Lung Center (UGMLC), Giessen, Germany

**Keywords:** Lung cancer, Neutrophils, A549 cells, Inflammation, Elastase, COX-2

## Abstract

The inflammatory tumor microenvironment plays a crucial role in tumor progression. In lung cancer, both bacterial infections and neutrophilia are associated with a poor prognosis. In this study, we characterized the effect of isolated human neutrophils on proliferation of the non-small cell lung cancer (NSCLC) cell line A549 and analyzed the impact of A549–neutrophil interactions on inflammatory mediator generation in naive and lipopolysaccharide (LPS)-exposed cell cultures. Co-incubation of A549 cells with neutrophils induced proliferation of resting and LPS-exposed A549 cells in a dose-dependent manner. In transwell-experiments, this effect was demonstrated to depend on direct cell-to-cell contact. This pro-proliferative effect of neutrophils on A549 cells could be attenuated by inhibition of neutrophil elastase activity, but not by oxygen radical neutralization. Correspondingly, neutrophil elastase secretion, but not respiratory burst, was specifically enhanced in co-cultures of A549 cells and neutrophils. Moreover, interference with COX-2 activity by indomethacin or the specific COX-2 inhibitor NS-398 also blunted the increased A549 proliferation in the presence of neutrophils. In parallel, a massive amplification of COX-2-dependent prostaglandin E_2_ synthesis was detected in A549–neutrophil co-cultures. These findings suggest that direct cell–cell interactions between neutrophils and tumor cells cause release of inflammatory mediators which, in turn, may enhance tumor growth in NSCLC.

## Introduction

Inflammatory cells and mediators of the tumor microenvironment may play a critical role in lung cancer progression. In 1863, Virchow detected leukocyte infiltration of neoplastic tissues and proposed a relationship between inflammation and cancer [1, 2]. Currently, the significance of this cancer-related inflammation has been outlined by defining inflammation as the seventh hallmark of cancer [3]. Although an established role in tumor progression has been attributed to macrophages and dendritic cells, the role of infiltrating neutrophils is less well defined [4–6].

Historically, neutrophils were exclusively regarded as part of the unspecific host defense against tumor cells. However, some recent studies gave evidence that tumor-associated neutrophils (TAN) may conversely play a decisive role in tumor progression.

Elevated systemic neutrophil counts [7] and high intratumoral leukocyte levels [8] have been identified as independent prognostic factors associated with a high relapse rate and a poor overall survival. Moreover, the intratumoral density of neutrophils has been shown to correlate with adverse prognostic factors such as elevated CRP levels [9]. In murine models of lung and pancreatic islet cell, cancer neutrophil depletion resulted in enhanced tumor growth under physiological conditions [10, 11]. Recently, it was proposed that the role of neutrophils in tumor biology is determined by their phenotypes, which may shift from an anti-tumor (N1-TAN) to a pro-tumor phenotype (N2-TAN) depending on cytokines of the tumor microenvironment [10, 12, 13]. The mechanisms leading to tumor promotion may include activation of the neutrophils` inflammatory potential such as the release of MPO and serine proteases from neutrophil granule contents [14–16]. Serine proteases may, on the one hand, induce tumor promotion by tissue degradation, thus facilitating invasion and spread of tumor cells [15]. On the other hand, in a murine model of lung adenocarcinoma, neutrophil elastase modified intracellular signaling pathways of tumor cells in a pro-tumorgenic way [16]. Beside the release of granule proteins, activated neutrophils release the reactive oxygen species superoxide anion (O_2_^−^) and hypochlorus acid. In lower concentrations, these substances may not be cytotoxic but genotoxic, thus promoting tumor progression [17].

In addition to releasing pre-stored secretory products, neutrophils synthesize arachidonic acid-derived lipid mediators, such as the 5-lipoxygenase-dependent leukotrienes and the cyclooxygenase (COX)-derived prostanoid prostaglandin E_2_ (PGE_2_). The inducible isoform of COX, COX-2, may be crucially involved in lung cancer pathogenesis: in vivo, COX-2 protein and mRNA levels are elevated and are associated with a poor outcome in lung adenocarcinoma [18, 19]. In vitro, over expression of COX-2 directly increases survival of lung adenocarcinoma cells lines [20].

Although strong evidence exists that infiltrating neutrophils play a decisive role in non-small cell lung cancer (NSCLC) progression [7–13], a direct pro-proliferative effect of isolated neutrophils on tumor cells in vitro has never been described. To mimic the interactions between neutrophils and NSCLC cells during pulmonary infection, we co-cultured freshly isolated human neutrophils with NSCLC cells of the human A549 adenocarcinoma cell line in the absence or presence of low doses of endotoxin (LPS). In essence, we found that neutrophils dose-dependently induce proliferation of unstimulated and LPS-exposed NSCLC cells, and the release of neutrophil elastase and COX-2 products were causally involved in this process.

## Materials and methods

### Isolation of human neutrophils

Neutrophils were isolated from venous blood of healthy donors by centrifugation over a Ficoll-Paque gradient (Pharmacia, Uppsala, Sweden). In brief, EDTA-anticoagulated blood was sedimented with 10 % dextran T 500 (Pharmacia) for 20 min. The neutrophil-containing supernatant was then layered over Ficoll-Paque and centrifuged at 400×*g* for 20 min. After removal of the mononuclear cell band, residual erythrocytes were removed by hypotonic lysis, cells were washed twice in Ca^++^/Mg^++^-free Hepes-buffered Hanks’ balanced salt solution (HHBSS−, no Calcium, no Magnesium, no phenol red, Gibco, Eggenstein, Germany), and finally resuspended in RPMI containing 1 % FCS at 10^7^ PMN/ml for proliferation experiments or in phenol red-free HHBSS containing Ca^++^ (1.25 mM)/Mg^++^ (0.5 mM) (HHBSS++, Gibco, Eggenstein, Germany) for the assessment of respiratory burst and elastase release.

### Flow cytometry

Purity of neutrophils was determined by flow cytometry analysis (BD FACSCanto, BD Biosciences, Heidelberg, Germany) using forward (FSC) and side (SSC) scatter characteristics and CD24 as neutrophil marker known to be expressed on mature neutrophils and on B lymphocytes. The cells were pelleted, resuspended in phosphate-buffered saline (PBS) containing 1 % bovine serum albumin (BSA), and incubated with a murine anti-human CD24 antibody conjugated to phycoerythrin (PE) and FITC-conjugated murine anti-human CD14-antibodies (BD Biosciences, Heidelberg, Germany) for 15 min. As negative control, murine anti-human immunoglobulins G_1_ (IgG_1_)–FITC/IgG_2_–PE (Simultest Control, BD, Heidelberg, Germany) were used. After the incubation period of 15 min in darkness, cells were washed again with 1 % PBS/BSA and were analyzed immediately using DIVA Software [21]. A total of 97 to >98 % of the isolated cells showed neutrophil FSC/SSC profiles and expressed CD24.

### Cell staining and viability

Additionally, neutrophil purity was confirmed by performing May–Gruenwald–Giemsa staining (Merck, Darmstadt, Germany). Staining revealed a purity of 96–97 % and showed that contaminating mononuclear cells amounted to <0.5 %. Cell viability of freshly isolated as well as of neutrophils cultured for 6 h in vitro was >96 %, as assessed by trypan blue dye exclusion.

### Cell culture

The A549 human lung adenocarcinoma cell line was obtained from the American Type Culture Collection (ATCC, Rockville, MD, USA) and cultured at 37 °C in a humidified atmosphere (95 % air, 5 % CO_2_). A549 cells were kept in Dulbecco’s modified Eagle’s medium (DMEM/F12, Gibco, Eggenstein, Germany) supplemented with 10 % fetal calf serum (FCS, Greiner, Frickenhausen, Germany) 2 mM l-glutamine, 10^5^ U/l penicillin, and 100 mg/l streptomycin. Cells were grown to confluence and subcultured every 2–3 days, at a split ratio of 1:10. Cell viability of A549 cells in culture was regularly assessed by trypan blue dye exclusion and was always >97 %.

Cell culture plasticware was purchased from Falcon (Mannheim, Germany).

### Neutrophil/A549 co-culture for the assessment of A549 proliferation and PGE_2_ release

The co-culture experiments were performed in 24-well cell culture plates (1 ml/well) at 37 °C in a humidified atmosphere (95 % air, 5 % CO_2_). A549 cells were plated at a density of 10^5^/ml in modified DMEM/F12. After 24 h, medium was harvested, and cells were incubated in 1 ml RPMI supplemented with 1 % FCS or in 1 ml HHBSS++ (assessment of elastase and O_2_^−^ release). When indicated, neutrophils were directly added to the tumor cells at given densities (varying from 0.5–10 × 10^6^ PMN/ml). Co-cultures were continuously shaken to prevent aggregation of neutrophils. In selected experiments, neutrophils were not placed directly onto the tumor cells, but co-cultured with A549 in a transwell system (700 µl/300 µl lower: upper compartment, pore size 0.4 µm). When indicated, LPS was simultaneously applied to neutrophil addition. In neutralization studies, the unspecific COX-inhibitor indomethacin (100 µM, Calbiochem, La Jolla, CA, USA), the selective COX-2 inhibitor NS-398 (10 µM, Calbiochem, La Jolla, CA, USA), the elastase inhibitor AAPVCK (5 µM) or the oxygen radical scavenger SOD (10 µg/ml, Sigma, Deisenhofen, Germany) were given simultaneously to neutrophil addition.

### Neutrophil/A549 co-culture for the assessment of neutrophil elastase release and respiratory burst

The co-culture experiments were performed in 24-well cell culture plates (1 ml/well) at 37 °C in a humidified atmosphere (95 % air, 5 % CO_2_). A549 cells were plated at a density of 10^5^/ml. Cells were grown to confluence. Immediately before neutrophil addition, medium was harvested and cells were kept in HHBSS++. Freshly isolated neutrophils were directly added to A549 cells (7.5 × 10^6^/ml in HHBSS++) to a total volume of 1 ml/well. Co-cultures were continuously shaken to prevent aggregation of neutrophils.

### Neutrophil monocultures

Freshly isolated neutrophils (7.5 × 10^6^/ml) were incubated on 24-well cell culture plates in 1 ml RPMI supplemented with 1 % FCS (for the assessment of proliferation and PGE_2_ release) or in HHBSS++ at 37 °C in a humidified atmosphere (95 % air, 5 % CO_2_) for the assessment of neutrophil elastase release and respiratory burst. Neutrophils were continuously shaken to prevent aggregation. To exclude that neutrophils were activated by shaking, basal release of elastase and PGE_2_ was monitored compared to non-shaking conditions. Shaking did not activate neutrophils, as basal release of elastase was even lower under non-shaking conditions (0.46 vs. 0.75 U/ml, *p* < 0,05) and basal PGE_2_ release did not differ between the two groups (105 pg/ml under non-shaking and 117 pg/ml under shaking conditions, *p* = 0.54).

### A549 monoculture

The monoculture experiments were performed to assess the effect of PGE_2_ and elastase on A549 growth in the absence of neutrophils. For that purpose, A549 cells were plated at a density of 10^5^/ml in modified DMEM/F12 on 24-well cell culture plates (1 ml/well) at 37 °C in a humidified atmosphere (95 % air, 5 % CO_2_). After 24 h, medium was harvested, and cells were incubated in 1 ml RPMI supplemented with 1 % FCS. When indicated, cells were either sham-incubated (control) or exposed to PGE_2_ (500 pg/ml, Cayman Chemical, MI, USA) or human Elastase (40 nM, Innovative Research, MI, USA) for 6 h, and MTS assay was performed as described below.

### MTS assay

The MTS assay (CellTiter 96@ Aqueous One Solution Cell Proliferation Assay, Promega, Mannheim, Germany) quantifies the metabolic activity of cells. This assay is based upon the cleavage of the yellow 3-(4,5-dimethylthiazol-2-yl)-5-(3-carboxymethoxyphenyl)-2-(4-sulfophenyl)-2H-tetrazolium, inner salt (MTS) to purple formazan by metabolic active cells. The production of the colored formazan product is directly proportional to the number of viable cells in culture [22]. Based on these data, the MTS assay is widely used for the assessment of cellular proliferation. In brief, A549 cells were seeded on 24-well plated and maintained in culture for 24 h. Then, medium was exchanged to RPMI containing 1 % FCS, and neutrophils (0.5–10 × 10^6^ PMN/ml) were added to a total volume of 1 ml/well. When indicated, co-cultures were stimulated with LPS (0.1 µg/ml *E. coli* LPS 0111:B4, Sigma, Deisenhofen, Germany). As negative controls, A549 cells were incubated in the absence of neutrophils with or without endotoxin stimulation (controls). After 6 h of incubation, the neutrophil containing supernatant was removed, cells were washed three times, and A549 cells were supplied with fresh medium (RPMI with 1 % FCS); 75 µl of MTS solution was added to each well to a total volume of 500 µl, and plates were again incubated for 2.5 h at 37 °C. Absorbance was read at 490 nm, background readings were subtracted from the sample wells and data were expressed as percentage of controls (A549 cells without neutrophils in the absence or presence of LPS). All samples were run in triplicates and all measurements were performed twice after 2.5 h of incubation with the MTS reagent. All data were expressed as percentage increase in MTS activity compared to unstimulated cells (controls) which were set to 100 %. In pilot experiments, monocultures of neutrophils (0.5–10 × 10^6^ PMN/ml) were run in parallel to co-cultures as an additional internal control. They were incubated on 24-well plates in the absence or presence of LPS. After 6 h of incubation, the same washing procedures were performed as described above. Fresh medium containing 75 µl of MTS solution was added to each well to a total volume of 500 µl, and samples were again incubated for 2.5 h. No MTS activity of monocultured neutrophils was detected in these studies.

### Superoxide anion generation

Neutrophil O_2_^−^ generation was assessed as superoxide dismutase-inhibitable reduction of cytochrome *C* according to Cohen [23]. Monocultures of neutrophils or co-cultures of neutrophils with A549 cells were activated with the chemotactic peptide n-formyl-methionyl-leucyl-phenylalanine (fMLP, 1 µM) for 10 min in HHBSS++. Duplicate reaction mixtures containing 75 µM ferricytochrome in the presence or absence of 10 µg/ml superoxide dismutase were performed. Incubations were terminated by centrifugation at 4 °C at 1,200×*g*. O_2_^−^ release was quantified as relative extinction at 550 nm in an Uvicon Spectrophotometer.

### Release of elastase

Elastase enzyme activity was measured by monitoring the turnover of l-pyroglutamyl-l-propyl-l-valine-*p*-nitro-anilide at 405 nm according to the method described by Kramps [24]. For induction of elastase release, monocultures of neutrophils or co-cultures of neutrophils with A549 cells were activated with fMLP (1 µM) for 10 min in HHBSS++. Incubations were terminated by centrifugation at 4 °C at 1,200×*g*. The cell-free supernatant was harvested and analyzed for elastase activity in an Uvicon Spectrophotometer as described above.

### Release of PGE_2_

PGE_2_ was quantified in a commercial ELISA-system (R&D Systems, Wiesbaden, Germany) according to the manufacturer’s instructions and was expressed in pg/ml. For these experiments, A549 monocultures, PMN monocultures and co-cultures were activated with LPS (0.1 µg/ml) for 6 h. Culturing and stimulation of these cells was done in RPMI containing 1 % FCS in 24-well culture plates at a total volume of 1 ml. At the end of the incubation period, cell supernatants were harvested, cell debris was removed by centrifugation at 13,000×*g,* and samples were stored at −20 °C until further processing. All samples were performed as duplicates and each sample was measured twice.

### Statistics

Data were analyzed by linear models using R [25]. Residuals were checked for possible deviations from normal distribution and heteroscedasticity. Results given in the text are mean and 95 % confidence (95 % CI) intervals.

**Fig. 1:**
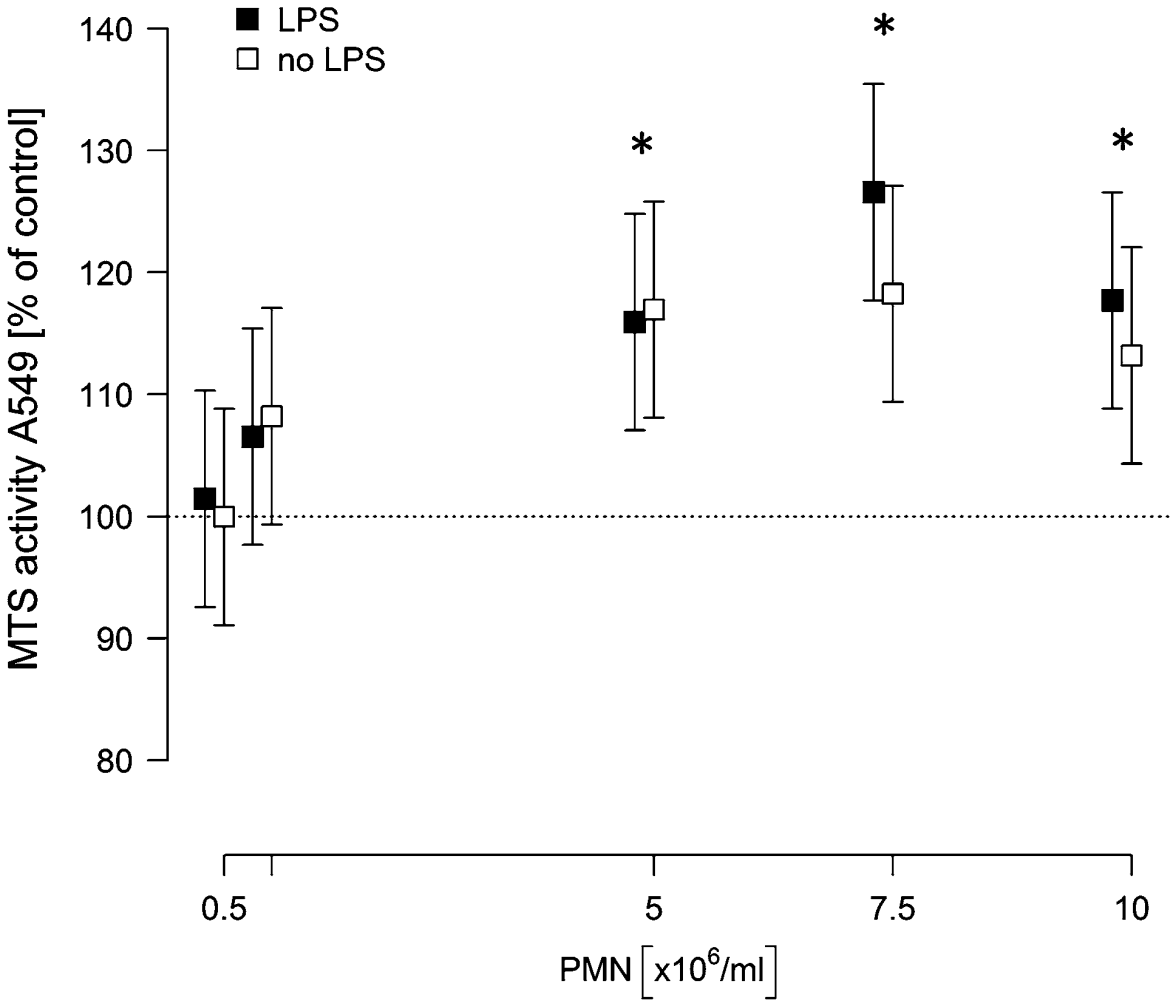
Neutrophil induce a dose-dependent proliferation of A549 cells. A549 cells were co-incubated with isolated neutrophils at given concentrations in the absence or presence of LPS [0.1 µg/ml] in a total volume of 1 ml. 6 h after incubation, the neutrophil-containing supernatant was removed, cells were washed three times, and 500 µl of fresh medium were added containing 75 µl MTS solution. After 2.5 h, absorbance was read at 490 nm. Values are expressed as percentage of MTS activity of A549 cells in the absence of neutrophils, which was set to 100 %. Means and 95 % confidence intervals of at least five independent experiments, each performed in triplicates, are given. *Asterisks* indicate *p* < 0.05 for the comparisons to the respective controls

For Fig. 1, several models were fitted. Dose dependency was modeled as MTS activity versus log PMN concentration for concentrations <10^7^ ml^−1^. In all other models, the PMN concentration was used as categorical predictor.

## Results

### Neutrophils induce proliferation of NSCLC cells in a dose-dependent manner

A549 monolayers were incubated with increasing concentrations of neutrophils (0–10 × 10^6^/ml) in the presence or absence of 0.1 µg/ml LPS for 6 h. Both in naive and in LPS-stimulated co-cultures, neutrophils dose-dependently increased the proliferation rate of A549 cells, which is expressed as the percentage of MTS activity of A549 monocultures (Fig. 1). The pro-proliferative effect of neutrophils was observed in unstimulated as well as in LPS-stimulated co-cultures. For neutrophil concentrations of 5 × 10^6^ PMN/ml, the activity was increased by 16 % independent of the presence of LPS. However, at PMN concentrations above 5 × 10^6^/ml, the stimulatory effect was slightly more pronounced in the presence of LPS (27 vs. 18 % for 7.5 × 10^6^ PMN/ml and 17 vs. 13 % for 10 × 10^6^ PMN/ml).

Since maximum proliferation of A549 cells was induced by 7.5 × 10^6^ PMN/ml in the presence of LPS (increase by 27 %) (95 % CI 18…35, *p* < 0.001), all further experiments were performed under these conditions.

### Direct cell-to-cell contact between neutrophils and NSCLC cells is mandatory for proliferation

**Fig. 2:**
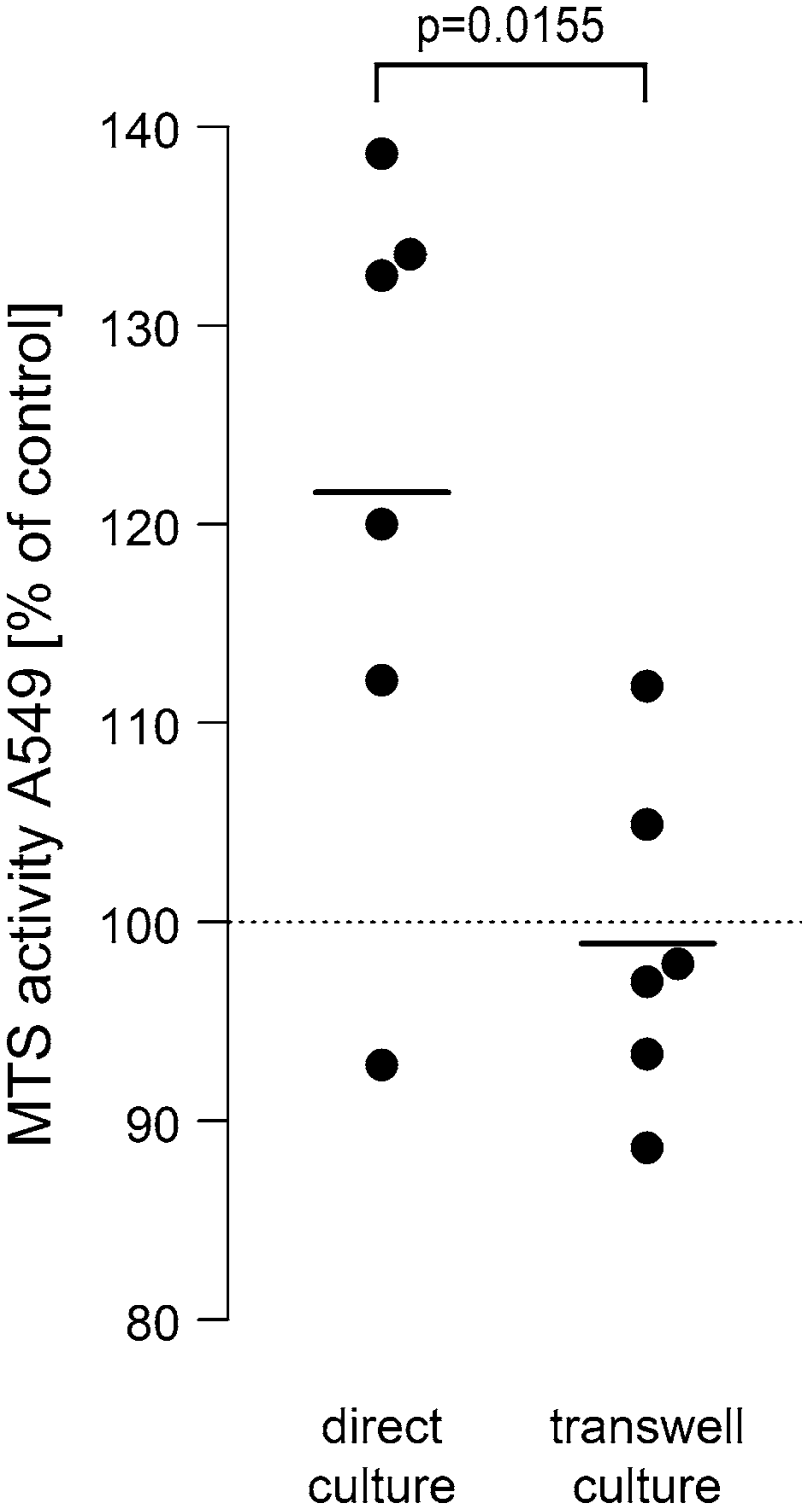
Direct cell-to-cell contact is a prerequisite for neutrophile-induced A549 proliferation. A549 cells were either directly co-incubated with neutrophils (7.5 × 10^6^/ml) or neutrophils were placed in the upper compartment of 0.4 µM pore transwells and co-cultured with A549 cells grown in the lower compartment of 24-well cell culture plates. All co-cultures were stimulated with 0.1 µg/ml LPS. After 6 h, the neutrophil-containing cell supernatant or the upper compartment were removed, A549 cells were washed three times, and again incubated for 2.5 h with 500 µl fresh medium containing 75 µl MTS solution. Absorbance was read at 490 nm. Values are expressed as percentage of MTS activity of A549 cells in the absence of neutrophils, which was set to 100 %, as indicated by the *horizontal dotted line*. *Horizontal bars* indicate averages of six independent experiments, each performed in triplicates

A549 monolayers were either directly incubated with 7.5 × 10^6^ PMN/ml or co-cultured in a transwell system with A549 cells seeded in the lower and neutrophils in the upper compartment. All cultures were treated with LPS (0.1 µg/ml). Interestingly, the pro-proliferative effect of neutrophils was suppressed when direct cell-to-cell contact was prevented under the given experimental conditions. In direct co-culture, MTS activity increased by 21.6 % as compared to −1 % in the transwell system (Fig. 2).

### Neutrophil elastase, but not oxygen radicals mediate the neutrophile-induced proliferation of A549 cells

**Fig. 3:**
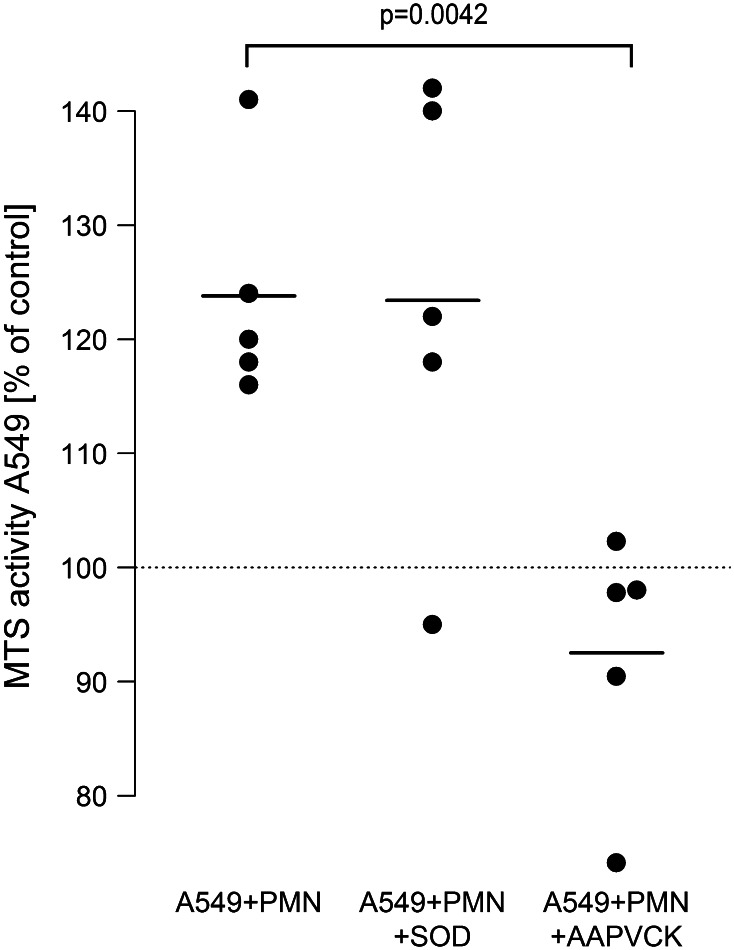
Neutrophil elastase, but not oxygen radical formation, is involved in neutrophil-induced A549 proliferation. LPS-activated [0.1 µg/ml] A549 cells were co-incubated with isolated neutrophils (7.5 × 10^6^/ml) in the absence (A549 + PMN) or presence of the oxygen radical scavenger SOD (10 µg/ml, A549 + PMN + SOD) or the elastase inhibitor AAPVCK (5 µM, A549 + PMN + AAPVCK). 6 h after incubation, the neutrophil-containing supernatant was removed, cells were washed three times, and 500 µl of fresh medium were added containing 75 µl MTS solution. After 2.5 h, absorbance was read at 490 nm. Values are expressed as percentage of MTS activity of A549 cells in the absence of neutrophils, which was set to 100 % as indicated by the *horizontal dotted line*. *Horizontal bars* indicate averages of five independent experiments, each performed in triplicates

To further elucidate the mechanisms in neutrophil-induced proliferation of A549 cells, neutrophil-derived inflammatory mediators were inhibited. In the presence of the highly specific inhibitor of neutrophil elastase AAPVCK, neutrophil-induced enhanced proliferation of A549 was prevented in the co-culture system (from 124 to 93 %). In contrast to this, neutralization of oxygen radicals by superoxide-dismutase had no detectable effect (Fig. 3). Addition of exogenous elastase (40 nM) to monocultures of A549 cells provoked an increase in proliferative activity to 115 % (95 % CI 110…121, *p* < 0.001).

**Fig. 4:**
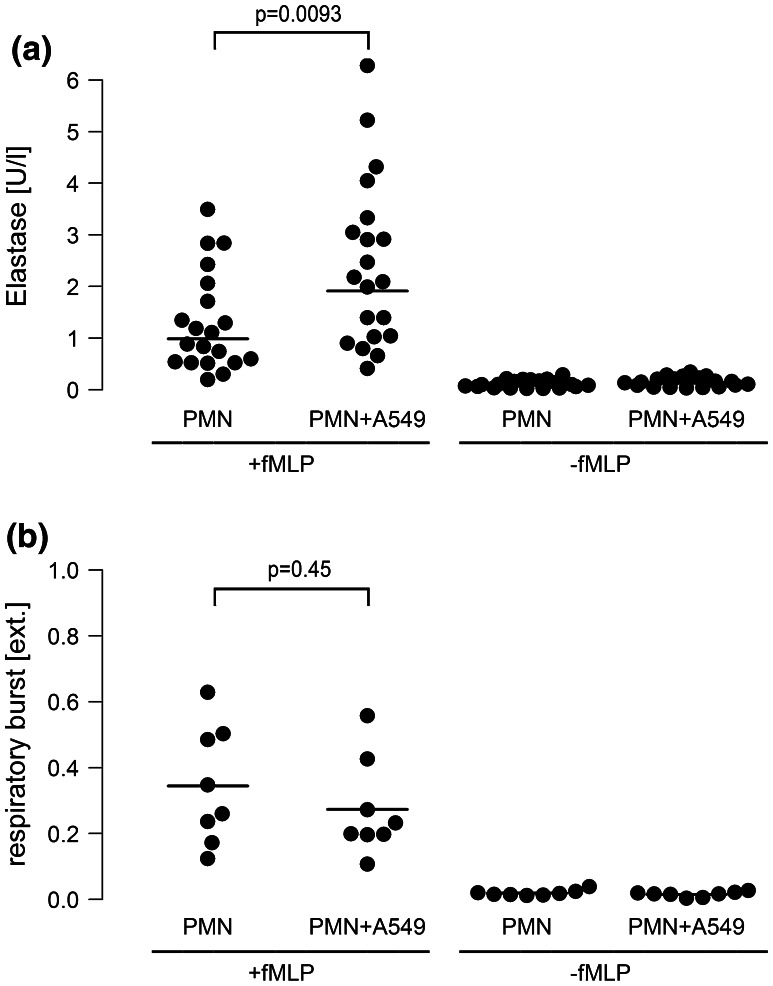
Release of neutrophile elastase, but not of oxygen radicals is increased in neutrophil-A549 co-cultures. Monocultures of neutrophils (7.5 × 10^6^/ml) or co-cultures of neutrophils (7.5 × 10^6^/ml) and A549 cells (PMN + A549) were activated with fMLP (1 µM). Experiments were performed in the presence of LPS [0.1 µg/ml]. **a** Elastase release: 10 min after fMLP stimulation, cell supernatants were collected and centrifugation was performed at 4 °C at 1,200×*g*. The cell-free supernatant was harvested and analyzed for elastase activity in an Uvicon Spectrophotometer. Horizontal bars indicate averages of twenty independent experiments. **b** Respiratory burst: duplicate reaction mixtures containing 75 µM ferricytochrome in the presence or absence of 10 µg/ml superoxide dismutase were performed. 10 min after fMLP stimulation, incubations were terminated by centrifugation at 4 °C at 1,200×*g*. O_2_^−^ release was quantified as relative extinction at 550 nm in an Uvicon Spectrophotometer. *Horizontal bars* indicate averages of eight independent experiments

Interestingly, fMLP-induced elastase secretion from neutrophils was doubled when co-cultured with A549 cells in the presence of LPS, while O_2_^−^ release from neutrophils remained almost unchanged (Fig. 4a, b). This corresponds well to the inefficiency of oxygen radical neutralization to attenuate proliferation of A549 cells.

### COX-2 activation is operative in PMN-mediated proliferation of A549 cells, and COX-2-derived PGE_2_ is massively amplified in neutrophil-A549 co-cultures

**Fig. 5:**
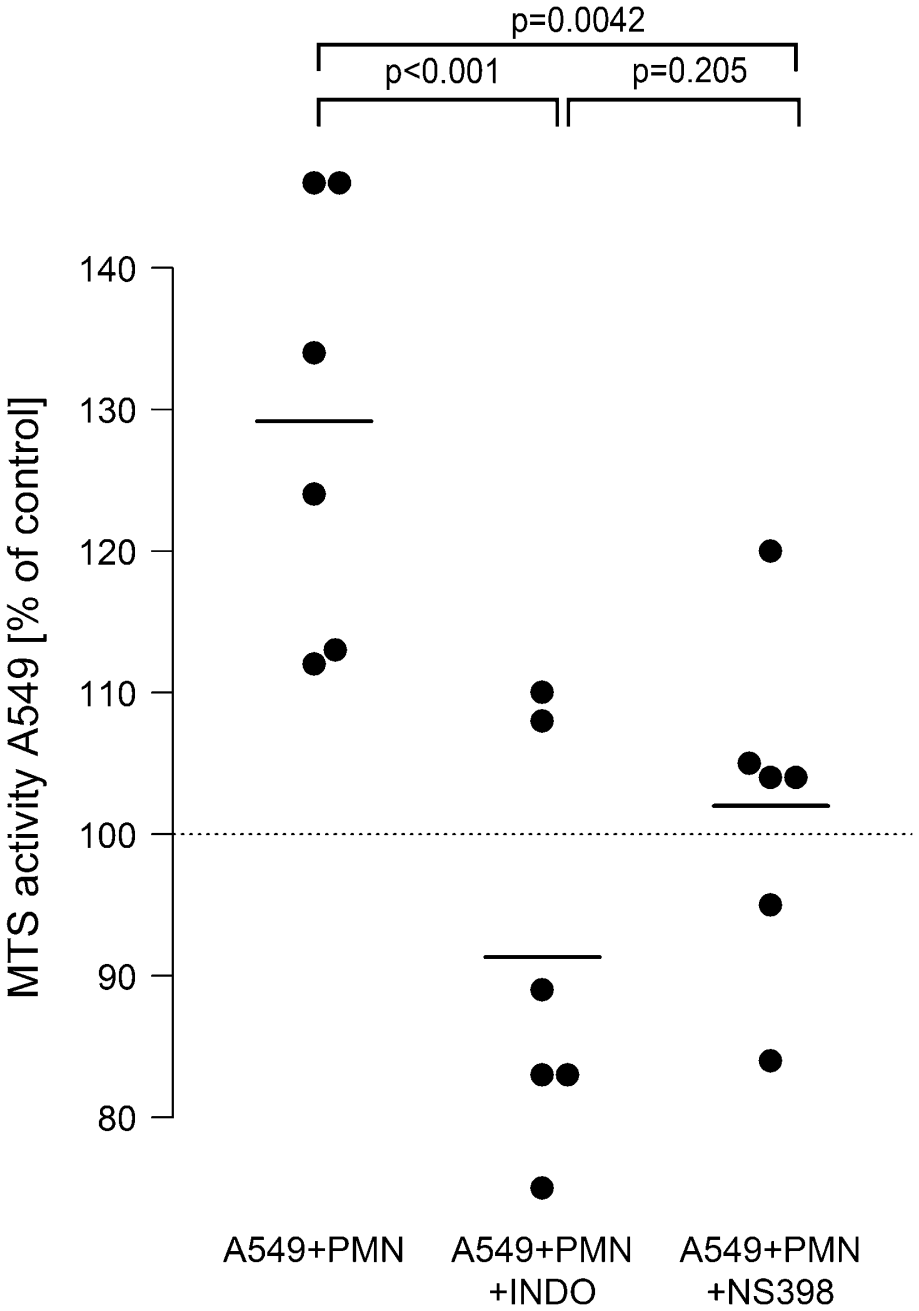
COX-2 activation is involved in neutrophil-induced A549 proliferation. LPS-activated [0.1 µg/ml] A549 cells were co-incubated with isolated neutrophils (7.5 × 10^6^/ml) in the absence (A549 + PMN) or presence of the unspecific COX-inhibitor indomethacin (100 µM) (A549 + PMN + INDO) or the selective COX-2 inhibitor NS-398 (10 µM) (A549 + PMN + NS398). 6 h after incubation, the neutrophil-containing supernatant was removed, cells were washed three times, and 500 µl of fresh medium were added containing 75 µl MTS solution. After 2.5 h, absorbance was read at 490 nm. Values are expressed as percentage of MTS activity of A549 cells in the absence of neutrophils, which was set to 100 % as indicated by the *horizontal dotted line*. *Horizontal bars* indicate averages of six independent experiments, each performed in triplicates

To evaluate the role of COX-2 activation, the role of this isoenzyme was defined in LPS-stimulated neutrophil-A549 co-cultures. In these experiments, A549 proliferation was raised to 129 %. Unspecific COX inhibition with indomethacin as well as specific interference with COX-2 by NS-398 completely blocked the pro-proliferative effect of neutrophils on A549 cells to basline levels. Average proliferation was 91 % in indomethacin-treated and 102 % in NS-398-treated co-cultures (Fig. 5).

**Fig. 6:**
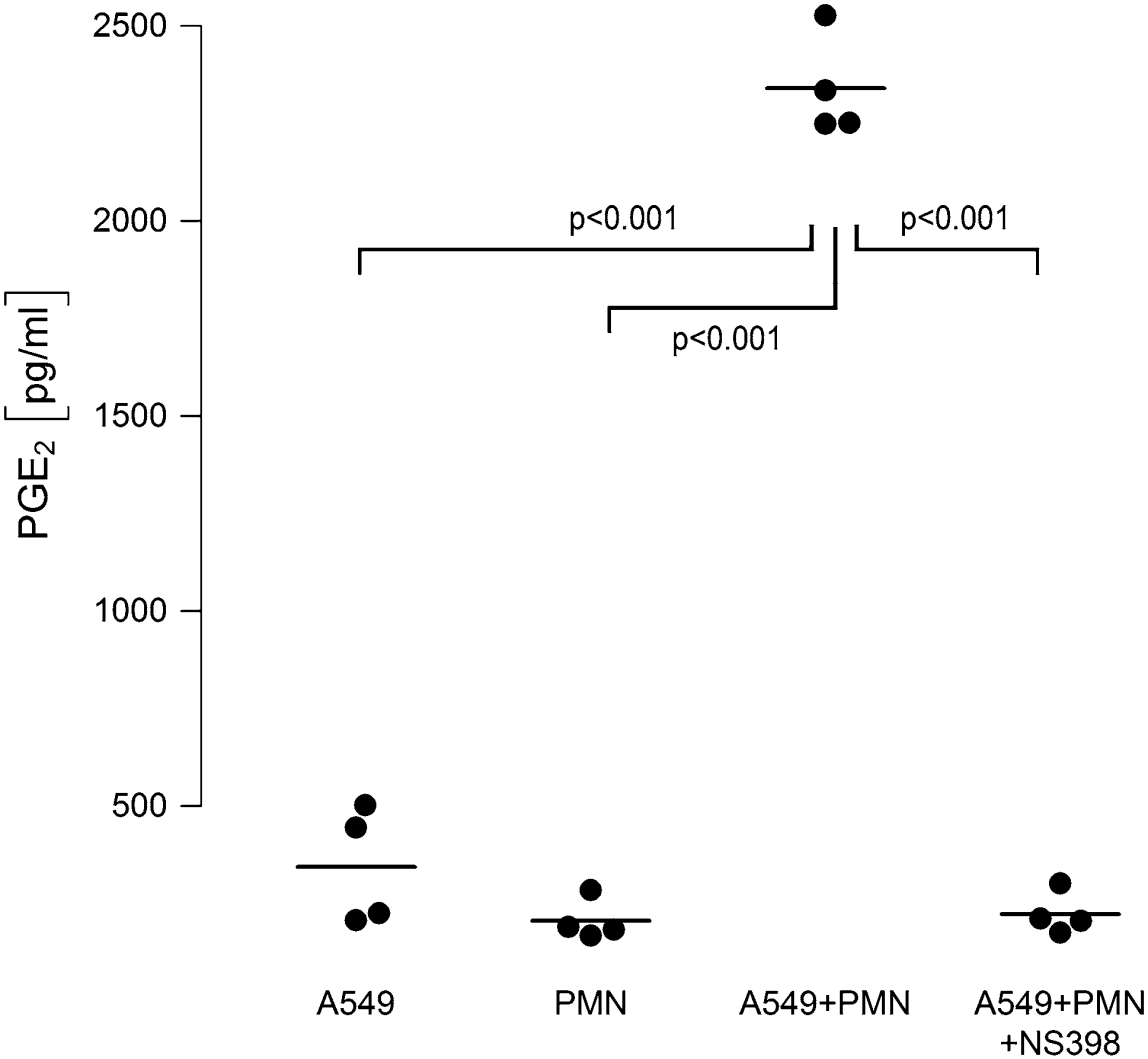
COX-2-dependent PGE_2_ release is amplified in A549–PMN co-cultures. Neutrophils (PMN, 7.5 × 10^6^/ml) and A549 cells were cultured separately and stimulated with LPS [0.1 µg/ml], or both cell types were kept in co-cultures and activated with LPS. Co-cultures were performed in the absence (A549 + PMN) or presence (A549 + PMN + NS398) of the COX-2 inhibitor NS398 (10 µM). After 6 h, cell supernatants were harvested and PGE_2_ synthesis was analyzed by ELISA. *Horizontal bars* indicate averages of four independent experiments, each performed in duplicates

Moreover, when analyzing cell supernatants for COX-2-derived lipid mediators, we found a nearly tenfold amplification of PGE_2_ in supernatants of co-cultured cells as compared to PGE_2_ released from monocultured neutrophils or A549 cells (Fig. 6). This amplification of PGE_2_ in neutrophil–A549 co-cultures was dependent on COX-2 activation, as PGE_2_ was down-regulated to levels found in monocultures in the presence of the non-specific COX-inhibitor indomethacin (not shown) and the specific COX-2 inhibitor NS-398 (Fig. 6). Interestingly, addition of exogenous PGE_2_ (500 pg/ml) induced an increase in proliferative activity of A549 monocultures to 121 % (95 % CI 108…134, *p* = 0.007) versus unstimulated controls.

## Discussion

The inflammatory tumor microenvironment plays a crucial role in promotion and progression of tumor growth. Although neutrophilia and neutrophil tumor infiltration are frequently encountered in solid tumors like NSCLC [26, 27], the role of neutrophils in tumor biology remains unclear. In the current study, addition of neutrophils to the adenocarcinoma cell line A549 enhanced proliferation of tumor cells. Proliferation of A549 cells in co-cultures was accompanied by a release of elastase and COX-2-derived PGE_2_, and inhibition of these mediators abolished tumor cell proliferation.

In our experimental setup, neutrophils dose-dependently increased the proliferation of A549 cells as quantified by an increase in MTS activity, which is directly proportional to cellular proliferation [22]. This increase in MTS activity was clearly related to A549 proliferation and not to co-incubated neutrophils. First, after 6 h of co-incubation, all neutrophils were removed by extensive washing procedures before the MTS assay was performed. Second, in pilot experiments, monocultures of neutrophils were run in parallel to co-cultures and no MTS activity was detectable in naive or LPS-stimulated PMN. Third, the increase in MTS activity in co-cultures was inhibited by antagonizing inflammatory mediators, such as COX-2-products, that are known to induce proliferation of A549 cells. And fourth, it has been shown that even when stimulated with proinflammatory agents, neutrophils have no proliferative capacity [28, 29]. Taken together, neutrophils exert a strong pro-proliferative effect on A549 cells in our experimental setup.

The effect of neutrophils on A549 proliferation was clearly dependent on the cell number of neutrophils used. The highest proliferation rate was elicited by 7.5 × 10^6^ PMN/ml. Greater neutrophil numbers failed to further enhance A549 proliferation. Given that proliferation of A549 was actually caused by neutrophil-derived mediators, this may, on the one hand, be explained by the fact that 7.5 × 10^6^ PMN/ml were sufficient to induce maximal A549 cell proliferation. On the other hand, we have previously shown that activity of neutrophils is closely regulated by their cell density and is down-regulated when cell density exceeds a critical number [30]. Whether the neutrophil concentrations currently used actually mimic those found in the tumor microenvironment in vivo cannot be deducted as only few studies address the neutrophil concentration in lung tumors in vivo [8]. However, the actual PMN/A549 ratio from ~30:1 was also chosen in other studies investigating the effect of isolated neutrophils on A549 biology [31].

In our experimental setup, the maximum proliferation of A549 cells was noted when PMN–A549 co-cultures were additionally exposed to low doses of endotoxin. This may relate to the phenomenon of “neutrophil priming.” Priming substances like cytokines or LPS render quiescent neutrophils more susceptible to secondary stimuli such as fMLP, bacterial exotoxins, or cell–cell contacts without activating them by themselves [32]. Such a priming effect may not only be operative in vitro, but may also be relevant in vivo. Lung cancer patients frequently suffer from gram-negative pulmonary infections, e.g., elicited by *E. coli* and *Haemophilus influenzae* [33–35], which contain LPS in their outer membrane. Therefore, the simultaneous exposure of lung cancer cells to neutrophils and LPS is very likely to occur in lung cancer patients.

Investigating the mechanism of neutrophil-induced A549 proliferation, we found that direct cell–cell contact between neutrophils and A549 cells was a prerequisite for amplified A549 proliferation in co-cultures. When A549 cells and neutrophils were cultured in transwells, which prevented direct cell–cell contact between the two cell types, enhanced proliferation of A549 cells was abrogated. We do not have an exact explanation for this phenomenon. However, neutrophils are capable to interact with A549 cells via binding of CD11/CD18 to ICAM-1 [36, 37], and ligation of ICAM-1 on A549 cells may directly activate intracellular signaling pathways (e.g., the MAP kinases ERK and JNK) with subsequent induction of cellular proliferation [38, 39]. Moreover, it has been shown that ligation of β_2_-integrins on neutrophils induces activation of their proinflammatory potential, including release of elastase and lipid mediators [40, 41], which were identified as strong promotors of A549 proliferation in our co-culture model. This might offer an attractive explanation for the absence of A549 proliferation when neutrophils and A549 cells were separated by transwells.

Neutrophil mediator release was another prerequisite for tumor cell proliferation. Release of neutrophil elastase, but not oxygen radical secretion, was essential for amplified A549 proliferation in our co-culture model. First, when elastase activity was blocked by the specific inhibitor AAPVCK [42], the amplified cell proliferation was substantially reduced, while neutralizing oxygen radicals by superoxide-dismutase was ineffective. Second, elastase release from neutrophils in co-culture with A549 cells was doubled, while the respiratory burst was unchanged in the co-culture model. And third, corroborating previous investigations, addition of purified neutrophil elastase to A549 monocultures was capable to induce proliferation of A549 cells in our study [16, 43]. Taken together, these findings suggest that neutrophil elastase is a key neutrophil-derived mediator which exerts strong proliferative effects on lung cancer cells. A mechanism of elastase-induced cell proliferation may include phosphatidylinositol-3 kinase hyperactivity and subsequent interaction with autocrine growth factor systems such as the platelet-derived growth factor and its receptor as recently demonstrated by Houghton et al. [16].

Most interestingly, not only neutrophil-derived elastase but also COX-2-derived lipid mediators were crucially involved in the pro-proliferative effects in our model. In co-cultures, a remarkable amplification of PGE_2_ synthesis was detected in the supernatant, which exceeded ~fivefold the sum of PGE_2_ release from monocultures of A549 or neutrophils. When COX activity was blocked by the non-specific inhibitor indomethacin or the COX-2-specific inhibitor NS-398, the release of PGE_2_ and the proliferation of A549 were normalized to baseline values. Thus, in LPS-stimulated PMN–A549 co-cultures, the cellular interaction leads to activation of COX-2 as indicated by amplified PGE_2_ release and increased tumor cell proliferation. The cellular source of PGE_2_ formation in the co-culture system remains to be elucidated. LPS is known to activate phospholipase A_2_ with subsequent release of free arachidonic acid (AA) from PMN [44]. Adjacent A549 cells may internalize and metabolize AA by COX-2 to PGE_2_. This transcellular eicosanoid metabolism has been detected in co-cultures of neutrophils and alveolar macrophages [45] and in knock-out mice [46]. Whether PGE_2_ is the decisive COX-2 mediator in inducing cell proliferation in our model cannot be conclusively derived from our data. However, PGE_2_ is the predominant COX product in lung cancer tissue [47], and addition of exogenous PGE_2_ to A549 monocultures strongly promoted cellular proliferation in the current study, thus confirming previous investigations [48]. Moreover, in preliminary experiments from our laboratory, inhibition of the PGE_2_ receptor EP2 effectively blocked LPS-induced A549 proliferation (Hattar et al., unpublished observations).

It is noteworthy, that both, inhibition of elastase activity and COX-2 activation equally reversed the PMN-induced tumor cell proliferation. These findings might be related to the previously described relationship between COX-2 activation and elastase release in neutrophils, with COX-2 inhibitors suppressing release of elastase [49]. Vice versa, neutrophil elastase has also been shown to stimulate PGE_2_ release from bronchial epithelia by activating COX-2 [50]. Thus, elastase and COX-2 may synergize to induce A549 proliferation in the co-cultures used.

In conclusion, our study demonstrates that neutrophils are capable of inducing A549 cell growth in a co-culture model. Neutrophil elastase release as well as COX-2 activation are both amplified by neutrophil–A549 cell interactions and are proven to be key regulators of A549 proliferation. Thus, these results shed light on the significance of neutrophils and inflammatory mediators of the microenvironment of tumors.

## Abbreviations

AA: Arachidonic acid
BSA: Bovine serum albumin
COX: Cyclooxygenase
FCS: Fetal calf serum
fMLP: *n*-Formyl-methionyl-leucyl-phenylalanine
FSC: Forward scatter
HHBSS: Hepes-buffered hanks’ balanced salt solution
LPS: Lipopolysaccharide
MTS: 3-(4,5-Dimethylthiazol-2-yl)-5-(3-carboxymethoxyphenyl)-2-(4-sulfophenyl)-2H-tetrazolium, inner salt
NSCLC: Non-small cell lung cancer
O_2_^−^: Superoxide anion
PE: Phycoerythrin
PGE_2_: Prostaglandin E_2_
PMN: Polymorphonuclear leukocyte
SSC: Side scatter
TAN: Tumor-associated neutrophils
